# Comparative Analysis of Dry and Wet Porometry Methods for Characterization of Regular and Cross-Linked Virus Removal Filter Papers

**DOI:** 10.3390/membranes9010001

**Published:** 2018-12-20

**Authors:** Simon Gustafsson, Frank Westermann, Tobias Hanrieder, Laura Jung, Horst Ruppach, Albert Mihranyan

**Affiliations:** 1 Division for Nanotechnology and Functional Materials, Department for Engineering Sciences, Uppsala University, Box 534, SE-751 21 Uppsala, Sweden; simon.gustafsson@angstrom.uu.se; 2 Charles River Biopharmaceutical Services, Gottfried Hagen Str. 20, 51105 Köln, Germany; frank@westermann.co (F.W.); tobias.hanrieder@crl.com (T.H.); laura.jung@crl.com (L.J.); horst.ruppach@crl.com (H.R.)

**Keywords:** gas sorption porometry, liquid-liquid porometry, cryoporometry, mille-feuille filter, parvovirus, virus removal filter

## Abstract

Pore-size distribution (PSD) is the most critical parameter for size-exclusion virus removal filters. Yet, different dry- and wet-state porometry methods yield different pore-size values. The goal of this work is to conduct comparative analysis of nitrogen gas sorption (NGSP), liquid-liquid and cryoporometry with differential scanning calorimetry (CP-DSC) methods with respect to characterization of regular and cross-linked virus removal filter paper based on cellulose nanofibers, i.e. the mille-feuille filter. The filters were further characterized with atomic force and scanning electron microscopy. Finally, the removal of the worst-case model virus, i.e. minute virus of mice (MVM; 20 nm, nonenveloped parvovirus) was evaluated. The results revealed that there is no difference of the obtained PSDs between the wet methods, i.e. DSC and liquid-liquid porometry (LLP), as well as no difference between the regular and cross-linked filters regardless of method. MVM filtration at different trans membrane pressure (TMP) revealed strong dependence of the virus removal capability on applied pressure. It was further observed that cross-linking filters showed enhanced virus removal, especially at lower TMP. In all, the results of this study highlight the complex nature of virus capture in size-exclusion filters.

## 1. Introduction

Virus removal filtration is a robust method to clear viruses and other microbial pathogens from liquids during manufacturing of protein-based pharmaceuticals and in water purification [1]. The first virus removal filters, which were based on graded nitrocellulose membranes, were developed by Elford in the 1930s [2,3,4,5,6,7]. Already then it was recognized that virus removal filtration is a complex process which can involve several simultaneous mechanisms, i.e. size exclusion (sieving), entrapment (depth filtration), interception (electrostatic or hydrophobic adsorption), and blockage (aggregation due to colloidal instability). When the size of the particle is larger than the size of the largest pore, true size-exclusion is the dominating mechanism of filtration. Most of the virus removal filters are not isoporous and have a certain pore-size distribution, sometimes done deliberately in order to enhance the flux across the filter. Therefore, virus breakthrough may potentially occur even when the nominal size of the pores is smaller than that of the virus. In the predominant majority of cases, the virus removal mechanism is highly complex and cannot be reduced to simple sieving/screening.

The most critical parameter for a size-exclusion filter is its pore-size distribution. Yet, different methods relying on various physical principles yield different pore-size values. This is because each method reflects the accessibility of pores to specific probing medium based on different assumptions. Normally, viruses are particles having a particle size between 20 and 200 nm, and therefore methods that can probe pores in this size range are particularly interesting for filtration applications. For the majority of mesoporous materials, i.e. materials having pores between 2 and 50 nm, nitrogen gas sorption porometry (NGSP) is the method of choice. The Barrett-Jonyer-Halenda (BJH) model, based on Kelvin equation, is applicable for NGSP and relates the pore-size distribution to emptying of pores from condensed liquid nitrogen as a function of surrounding nitrogen relative pressure. NGSP is an isothermal method performed on dry samples. However, from the point of filtration, application methods probing the pore-size distribution in the wet state are preferable. In this respect, two wet-porometry methods that are useful to probe mesoporous materials include liquid-liquid porometry (LLP) and cryoporometry with differential scanning calorimetry (CP-DSC). LLP is an isothermal method that is based on replacement of one pore-filling liquid with another immiscible liquid, wherein the pore size is related to applied pressure needed to replace two liquids with each other based on the Hagen-Poiseuille equation [8,9]. CP-DSC is an isobaric method, which relates the pore size to the melting pressure depressing of water present in pores as compared to bulk water situated outside the pores [10].

Another parameter that can affect the efficiency of virus removal is the applied transmembrane (TM) pressure during filtration [11,12,13,14]. Already in the 1930s, Elford and Ferry observed anomalous behavior during filtration when an isoelectric serum albumin could readily diffuse through 14 nm nitrocellulose filter, but it would be impermeable through 45 nm membrane filtered at 3 bar pressure [7]. The latter implies that irrespective of the pore-size characterization method used, it is important to test the filter under different processing conditions that are close to real application using worst-case model viruses.

In this work, we focus on the characterization of the pore-size distribution and separation properties of a new type of nonwoven virus removal filter paper developed at Uppsala University, Sweden. The filter paper consists of 100% nanocellulose and is produced by hot-pressing technology, similar to papermaking, rather than by phase inversion that is utilized for membrane manufacturing [15,16,17,18,19]. The filter was shown effective in removal of swine influenza A virus [18], xenotropic murine leukemia virus [15], and minute virus of mice [16] with log10 reduction values >5. The mille-feuille filter further shows high protein throughput and low tendency to fouling thanks to the innate hydrophilicity of cellulose nanofibers [20]. The filter is different from other known virus removal filters owing to its unique internal architecture consisting of stacks of self-assembled 2D-nanocellulose sheets producing a so-called “thousand leaves” (mille-feuille) structure [16,17]. It also features a relatively high overall porosity, i.e. 35–40% as well as low levels of potential leachables and extractables [18,19,21]. Furthermore, it has been shown that the wet strength properties of the virus removal filter paper can be significantly improved by cross-linking [19].

The goal of this article is to perform comparative analysis of various dry- and wet-porometry methods in the context of worst-case model virus, i.e. minute virus of mice (MVM), filtration.

## 2. Material and Methods

Based on the previous results, 33 μm filters were used for this study as they provide a relatively good balance between virus removal, mechanical stability, and flux performance [15].

### 2.1. Filter Preparation

A 3 wt% Cladophora cellulose dispersion was prepared in deionized water using a high-energy ultra-sonicator (750 W; 20 kHz; 13 mm probe; Vibracell; Sonics, Newtown, CT, USA) using 30 s pulses at 70% amplitude for 20 min. To cast the filter, 50 mL of the Cladophora cellulose dispersion was added to 300 mL of water. The dispersions were then drained over a 0.65 μm filter membrane (Durapore^®^, Merck Millipore, Burlington, MA, USA) using vacuum suction in a funnel. The cellulose cake on the filter was then removed and dried at 40 °C for 12 h using a hot-press (Rheinstern; Mainz, Germany).

### 2.2. Cross-Linking of Filter

The esterification cross-linking between the citric acid and the hydroxyl groups on the cellulose catalyzed by sodium hypophosphite was conducted as described earlier [19,22,23]. A solution containing 0.5 g of sodium hypophosphite monohydrate and 0.4 g of citric acid in 50 mL of deionized water solutions was prepared. The cross-linking solution was then added to the funnel just before draining stage as described above. The wet cellulose cake was then dried at 80 °C for 12 h.

### 2.3. Scanning Electron Microscopy

A Zeiss Merlin scanning electron microscope (SEM system, Zeiss, Oberkochen, Germany) was used. The images was obtained using 0.8 kV acceleration voltage, and the sample was sputtered with Pd/Au prior to the analysis to limit electrostatic charging effects.

### 2.4. Atomic Force Microscopy

A Bruker Dimension Icon atomic force microscopy (AFM, Bruker, Billerica, MA, USA) system with a Bruker silicon nitride ScanAsyst-Air probe (Bruker, Billerica, MA, USA) was used to obtain the images. The probe has a symmetric pyramid geometry with a nominal tip radius of 2 nm. The filter was mounted using a double adhesive tape on magnetic disc holders. Bruker’s ScanAsyst software (Version 1.2, Bruker, Billerica, MA, USA) was used with the instrument running in peak-force taping mode to obtain the images.

### 2.5. Nitrogen Gas Sorption Analysis

Nitrogen gas sorption isotherms were obtained using ASAP 2020 instrument (Micromeritics, Norcross, GA, USA). The performance of the instrument was validated using Micrometrics^TM^ Silica-Alumina SSA 210 m^2^·g^−1^ (lot number: A-501-49) standard prior to analysis. The deviation between the pore-size mode of the calibration data from the nominal standard values was 0 nm. The sample was degassed for 6 h at 95 °C at 5 °C·min^−1^ ramp temperature prior to analysis. The pore-size distribution was calculated according to the Barrett-Jonyer-Halenda (BJH) method [24] from the desorption branch of the isotherm. Here and throughout the text, pore-size mode refers to the position of the highest peak.

### 2.6. Liquid-Liquid Porometry (LLP)

The liquid-liquid displacement method was used as proposed by Erbe [25] and Bechhold et al. [26]. This method is a standard industrial method used for characterizing filters in the wet state under the close-to-operational conditions, including virus removal filters, e.g., Viresolve by Merck Millipore [9]. Two immiscible liquids were used for LLP, i.e. sulphate-rich intrusion liquid (25% ammonium sulphate, 0.04% polyethylene glycol (PEG) 8000, and 75% water) and PEG-rich wetting fluid (40% ammonium sulphate, 59% PEG 8000, and 1% water). The mille-feuille filter (45 mm in diameter) was placed in a stirred cell holder with underlying general-purpose filter paper support. The rate of flow was monitored gravimetrically by collecting the outflowing liquid on an analytic balance, connected to LabX V1.5 software (Mettler Toledo, Columbus, OH, USA), at 10 s intervals. The wetting fluid was passed in the stirred cell and forced through the mille-feuille filter at a pressure of 5.5 bar for 15 min in order to fill all pores. The procedure was repeated until steady curve was observed. Then, intrusion liquid was added in the container. The overhead pressure was increased gradually with 0.1 bar increments at 15 min intervals, and the flow of intrusion liquid was monitored as described above. The pressure at which the flow of intrusion liquid is registered first corresponds to the size of the largest pore in the filter. The reference flux of the intrusion liquid was estimated as a linear extrapolation from the flux values at 4 and 5 bars obtained from the measurements. Equivalent pore radius was calculated using Laplace Equation:

(1) $$r=\frac{2\gamma kcos\theta }{∆P}$$

where *k* is a shape factor, set to 1 with the assumption of cylindrical pores, *γ* is the surface tension, *θ* is the contact angle between the interface and the pore wall, Δ*P* is the TM pressure and *r* the effective radius.

The flow weighted pore-size distribution was calculated using Equations (2) and (3) as described by S. Giglia et al. [9]:

(2) $$R\left(∆P\right)=\frac{Q_{2phase}\left(∆P\right)}{Q_{Intrusion\;fluid}\left(∆P\right)}$$

(3) $$F_{Q}\left(r\right)=\frac{d\left(R\left(∆P\right)\right)}{d\left(∆P\right)}\;\cdot \;\frac{∆P^{2}}{2k\gamma cos\theta }$$

where *Q*_2*phase*_(Δ*P*) is the volumetric flow of intrusion liquid when the wetting fluid is present in the membrane at a set pressure, *Q_Intrusion fluid_*(Δ*P*) is the volumetric flow of intrusion fluid in absence of wetting fluid present in the membrane, Δ*P* is the pressure, *F_Q_*(*r*) is the flow weighted pore-size distribution, *k* is the shape factor which is set to 1 approximating a cylindrical pore shape, and *γ* is the interfacial surface tension between the two fluids, which has been reported as approximately 6.3 × 10^−4^ N at 22 °C [27]. The measured volumetric two-phase flow data were fitted to a weighted smooth spline function using Matlab. Equation (3) was used with a step size increment of 2 nm to obtain the weighted flow fraction bar plot, a Gaussian function was then fitted to the histogram in order to visualize a probable pore-size distribution.

### 2.7. Cryoporometry by Differential Scanning Calorimetry

The CP-DSC method was used as proposed by Landry [10]. The samples were cut into small 1 mg pieces and soaked in deionized water for 2 h. Before placing into the crucible with lid, the excess water on the filter sample was removed by lightly touching every piece on a paper towel, then 2 mg of the sample was added into preweighted aluminum crucibles. All crucibles were sealed and weighed. Mettler DSC 3 instrument equipped with autosampler was used for analysis. The sample was first frozen at 10 K·min^−1^ rate to −20 °C and then heated to 5 °C at a heating rate of 0.7 K·min^−1^. 

For a porous material with a narrow pore-size distribution, the melting point depression can be related to a pore radius using Equation (4):

(4) $$∆T_{on-pk}=\frac{A_{m}}{r-\delta_{m}}+B_{m}$$

where ∆*T_on-pk_* is the difference of the depressed melting point to the true melting point of bulk water, r is the radius of the pore. *A_m_*, *B_m_* and *δ_m_* are probing liquid dependent constants. Herein, we use the following values for *A_m_* = 19.082, *B_m_* = −0.1207, and *δ_m_* = 1.12 calculated by Landry [10]. The differential pore volume (d*V*/d*r*) was calculated using Equation (5):

(5) $$\frac{dV}{dr}=\frac{dQ}{dt}\cdot \frac{dt}{d\left(∆T\right)}\cdot \frac{d\left(∆T_{on-pk}\right)}{dr}\cdot \frac{1}{m\cdot ∆H_{f}\left(T\right)\cdot \rho \left(T\right)}$$

where d*Q*/d*t* is the heat flow, d*t*/d(∆*t*) is the scanning rate of 0.7 K/min, d(∆*T_on-pk_*)/dr is the melting point depression from Equation (4), m is the mass of the sample, ∆*H_f_*(*T*) is the temperature-dependent melting enthalpy, and *ρ*(*t*) is the temperature-dependent density.

The temperature-dependent melting enthalpy was calculated using Equation (6):

(6) $$∆H_{f}\left(T\right)=334.1+2.119\left(T-T_{m}^{0}\right)-0.00783{\left(T-T_{m}^{0}\right)}^{2}$$

where *T* is the current temperature and *T_m_*^0^ is the equilibrium melting temperature of water.

The temperature-dependent density of water was estimated using Equation (7):

(7) $$\rho \left(T\right)=-7.1114+0.0882T-3.1959\times 10^{-4}T^{2}+3.8649\times 10^{-7}T^{3}$$

where *T* is the temperature in Kelvin.

To achieve peak separation between bulk water and pore-confined water peaks, a second-degree Gaussian function is fitted to the peak of the confined water using Matlab (*r*^2^ = 0.99). The estimated function is then used as d*Q*/d*t* in Equation (5) to calculate the pore-size distribution.

The melting temperature onset of bulk water, representing the water outside of the pores, was measured and calculated as follows. The DSC endotherm was measured for 5 different volumes of water viz. 1, 2, 4, 6, and 10 µl (*n* = 5 for each volume 0.7 K·min^−1^ heat rate) and the mean peak temperature was calculated to 0.61 ± 0.1 °C.

### 2.8. Flux Measurement

The mille-feuille filter (45 mm in diameter) was placed in a filter holder (Advantech KST-47) with underlying general-purpose filter paper support. The filter holder was filled with 200 mL deionized water. The rate of flow was measured for a set volume of 50 mL for each pressure viz. 1, 2, 3, 4, and 5 bars. The rate of flow was monitored gravimetrically by collecting the outflowing liquid on an analytic balance, connected to LabX software (Version 2.5, Mettler Toledo, Columbus, OH, USA), at 20 s intervals. All measured values were normalized per bar in order to visualize the compaction effect of the filters.

### 2.9. Virus Removal by EPT and LVP

Virus spiking tests were performed similar to the flux measurement as described. The filter holder was filled with 200 mL of 0.1% bovine serum albumin in phosphate buffer solution, spiked with MVM. Five fractions of 40 mL each were collected during the filtration experiment. The virus titres before and after filtration were evaluated using end-point titration (EPT) and large-volume plating (LVP) methods. The MVM titres were evaluated from observed cytopathogenic effect (CPE) and tissue culture infectivity dose (TCID_50_) at 8-fold dilution. EPT was used for samples with high viral loads. LVP was performed for samples with expected low virus loads, provided that such indications had been demonstrated during EPT.

Prior to use, the A9 cells were checked for the correct phenotype, absence of contaminations (fungi or mycoplasma), and sufficient cell density. Upon adding of virus-containing solution, both EPT and LVP plates were incubated at 37 ± 1 °C and a CO_2_ concentration of 5.0 ± 0.5% by volume. After the first phase of the incubation, i.e. after 6–8 days, the supernatants were transferred onto fresh cell indicator MTPs and incubated for another 5–7 days. The total duration for the incubation phase was 11–15 days, enabling a clear evaluation of CPE.

**Figure 1:**
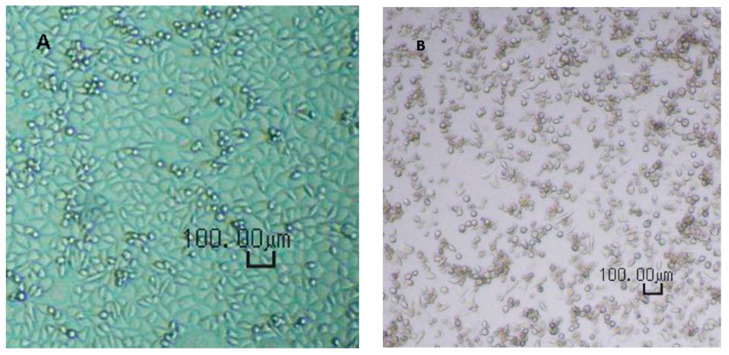
Cytopathogenic effect (CPE) illustrated for reference A9 cells incubated with or without minute virus of mice (MVM): (**A**) healthy cells and (**B**) infected cells. CPE: cytopathogenic effect; MVM: minute virus of mice.

The CPE was verified microscopically as shown in Figure 1.

Each individual well at varying dilutions was tested, and positive wells were documented. The results for EPT were then analyzed using the Spearman-Kärber formula (Equation (8)) as described previously [28,29].

(8) $$\frac{\log_{10}\left(TCID_{50}\right)}{ml}=-\left(-Y_{0}+\frac{d}{2}-d\sum P_{i}-v\right)$$

*Y*_0_: decadic logarithm of highest dilution factor of the sample, which causes the infection of all parallel cultures (= 8)

*d*: decadic logarithm of dilution step (= log_10_3);

*P_i_*: observed reaction rate starting and including the rate at *Y*_0_;

*v*: decadic logarithm of volume conversion factor (= log_10_5).

In the LVP assay, the lowest dilution is analyzed on multiple wells. With this, a higher total volume of the original samples is screened for residual infectivity.

If virus-induced changes in 15 to 50% of all wells of the LVP assay are observed, the virus titer is calculated according to the Spearman-Kärber formula (see above). It is assumed that a 1:3 higher concentrated dose compared to the dose analyzed leads to an infection of all parallel cultures. Only two reaction rates are reported: the reaction rate determined by the LVP and the reaction rate of the virtual 1:3 higher concentrated dose (= 1).

If virus-induced changes are observed in only a few wells of the LVP (<15% of all wells) for a sample, the virus titer is calculated according to Equation (9):

(9) $$\frac{\log_{10}\left(Titer\right)}{ml}=\log_{10}\left[\frac{D}{V_{w}}\left(-\ln\frac{n-n_{p}}{n}\right)\right]$$

*D*: predilution factor of the sample;

*n_p_*: number of virus-positive wells;

*n*: number of all wells tested;

*V_w_*: sample volume per well (0.2 mL).

If no virus-induced changes are observed for a sample, the virus titer is determined by the Poisson distribution (Equation (10)) at the 95% confidence limits:

(10) $$\frac{\log_{10}\left(Titer\right)}{ml}=\log_{10}[\frac{\ln p}{v\cdot \ln\left(1-\frac{v}{V}\right)}]$$

*p*: 0.05;

*v*: tested sample volume in mL;

*V*: process fraction volume in mL.

The reduction of viral load, expressed as a logarithmic reduction value (LRV), represents the capability of a virus clearance, calculated using Equation (11):

(11) $$LRV=\log_{10}\frac{C_{0}}{C_{n}}$$

*C*_0_: log_10_ total virus load spiked start material

*C_n_*: log_10_ total virus load related filtrate

The titer was measured for each fraction individually, and the confidence interval for each measurement point in the duplicate run was 95%.

## 3. Results and Discussion

**Figure 2:**
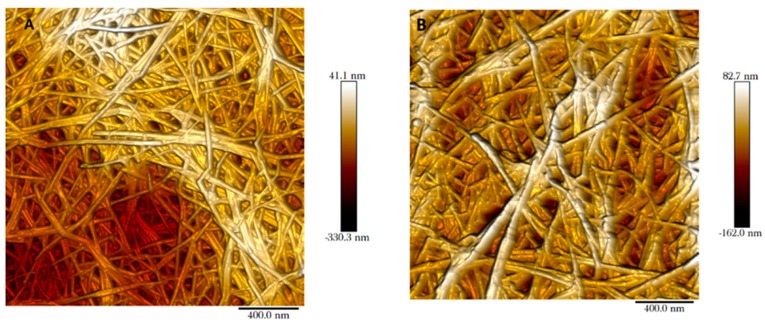
Atomic force microscopy (AFM) images of as-produced regular (**A**) and cross-linked (**B**) mille-feuille filter.

The mille-feuille filter is a nonwoven wet-laid filter paper produced by hot-pressing cellulose nanofibers. The pores in the filter paper are made up by the voids between cellulose nanofibers. These pores can be visualised through AFM imaging for both regular and cross-linked filters as shown in Figure 2. In this top-view AFM image, the cellulose nanofibers and interstitial voids are clearly visible. The randomly oriented cellulose nanofibers form a porous web-like structure in nm size range. Note that the interstitial space between cellulose nanofibers seen in the AFM images only reflects the properties of the outermost layer.

**Figure 3:**
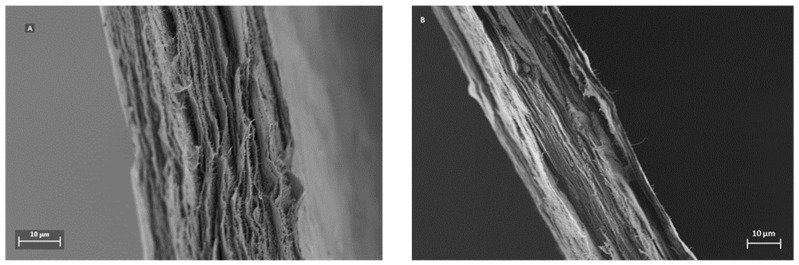
Cross-section scanning electron microscope (SEM) images of as-produced regular (**A**) and cross-linked (**B**) mille-feuille filter.

A distinctive feature for mille-feuille filter is its layered architecture, which can be visualized by SEM. Figure 3 shows the cross-section images of the filters, i.e. regular (A) and cross-linked (B). The unique stratified structure of mille-feuille filter can be clearly seen. The structure of the as-produced cross-linked filter seems more densely packed, as evidenced by diminished distance between each layer. In all, the top-view AFM and cross-section SEM images suggest the presence of different levels of porosity and thus anisotropic structure.

**Figure 4:**
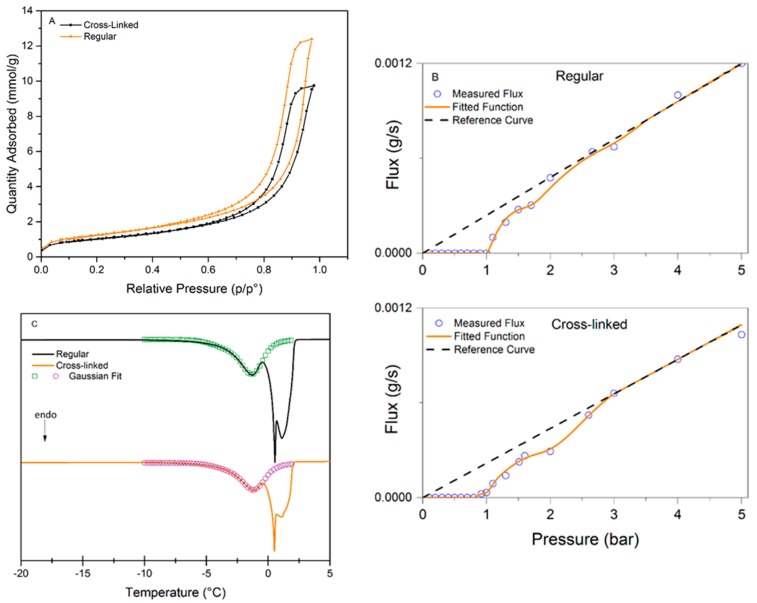
(**A**) Isotherm from nitrogen sorption measurement for both regular and cross-linked filter; (**B**) LLP isotherm for regular and for cross-linked filter; (**C**) Cryoporometry isobar for regular and cross-linked filter. The data within the PSD was fitted to a Gaussian distribution to obtain good peak separation. LLP: liquid-liquid porometry; PSD: pore-size distribution.

As it was mentioned earlier, the choice of the appropriate method for pore-size characterization is not straightforward. Figure 4 presents the analysis data for the three methods used to characterize the pore-size distributions of regular and cross-linked virus removal filter papers. Figure 4A shows the nitrogen sorption isotherms of the studied samples. Both samples exhibited a hysteresis, which suggests the presence of mesopores. It should be noted that the total pore volume decreased when the filter is cross-linked, which would suggest a more densely packed structure of the sample. Figure 4B presents the results of LLP isotherms. It is seen in this plot that the flux values in the cross-linked samples are slightly higher than in the regular one. The flow commenced at 1.00 ± 0.08 bar for the regular filter compared to 0.91 ± 0.08 bar for the cross-linked sample, indicating that the pores are larger or more accessible for the probing liquid in the cross-linked sample. Figure 4C shows the CP-DSC isobars of the studied samples. Two peaks are visible in both samples, the peak at subzero temperatures corresponds to water confined in the pores, and the second peak is nonconfined bulk water freezing. The peak in the heat flow curve is slightly smaller for the cross-linked sample, indicating lower enthalpy of melting, which may indirectly reflect smaller pore volume.

**Figure 5:**
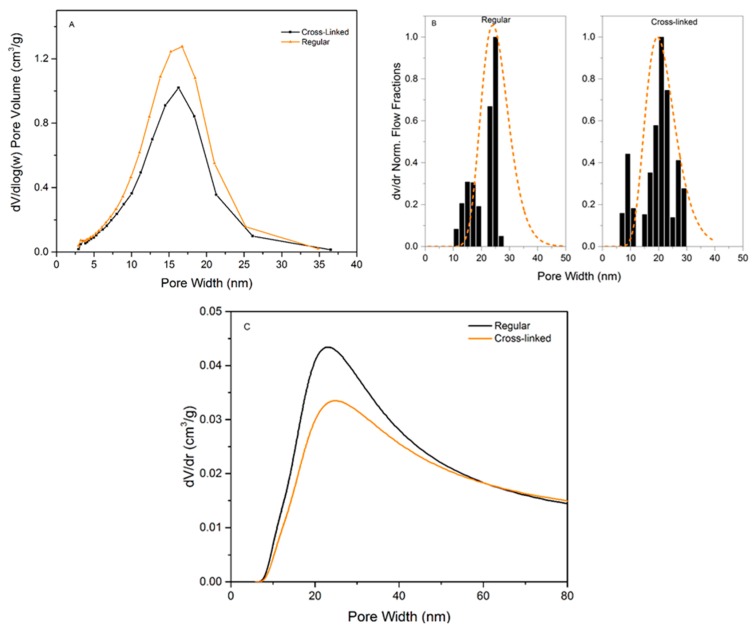
(**A**) Calculated pore-size distribution for regular and cross-linked filters from nitrogen desorption isotherm using the BJH method. (**B**) Calculated pore-size distribution for regular and cross-linked filters from LLP measurement data. The dotted line is a visual PSD guide for the eye obtained by fitting a lognormal distribution to the data. (**C**) Calculated pore-size distribution for regular and cross-linked filters using cryoporometry data.

The calculated pore-size distributions for the three methods can be seen in Figure 5 and the pore-size data are summarized in Table 1. Figure 5A shows the PSD from NGSP using NGSP model. Almost no difference between the regular and cross-linked filters is observed since the pore mode for each sample is centered at ~19.5 nm (regular 18.9 ± 1.4, cross-linked 20.0 ± 2.4 nm). However, the pore volume is lower for the cross-linked sample, i.e. 0.34 g/cm^3^ for the cross-linked compared to 0.43 g/cm^3^ for the regular filter. Based on the SEM images of cross-sections, we hypothesize that cross-linking reduces the space between individual nanocellulose sheets. Figure 5B shows the PSD from LLP data. The analysis of the LLP data produces a multimodal size distribution, which highlights the difficulty of measuring very low flows with high accuracy. However, there is no statistical difference between the pore modes, i.e. 24.3 ± 2.5 nm for the regular and 22 ± 1.4 nm for the cross-linked sample. Even though the flow commenced at a lower pressure for the cross-linked sample, the model reveals that the higher increase of flux at ~1.2 bars compared to the regular filter shifts the pore mode towards a smaller diameter. Figure 5C shows the PSD obtained from CP-DSC analysis. The pore mode of 24.0 ± 0.8 nm is assessed for both the regular and 24.0 ± 0.6 nm for the cross-linked filters. Similar to NGSP analysis, the pore volume is slightly lower for the cross-linked sample.

In general, it is concluded that there is not a significant difference between the methods regarding detected pore size. It should be noted that the NGSP and LLP managed to detect the upper pore size cut-off around 35–40 nm, which is of importance for virus removal filtration. From the point of practical utility, the LLP method was less practical to implement compared to the CP-DSC method. Firstly, a larger sample was required per test due to the size of the suitable filter holder. Secondly, the procedure was performed manually and, therefore, it was laborious and time-consuming. Because of the large sample size, the method further required relatively large quantities of liquids with specific composition and viscosity. In general, higher variability of data was observed compared to the other automated methods. Contrary to LLP, CP-DSC method was preferable because it was automated and did not require large sample size or large volumes of liquid. As a result, the produced data showed less variability than that seen with LLP. Among the drawbacks of the CP-DSC, the uncertainty related to determination of the freezing point for bulk water needs to be highlighted. Another drawback is the poor peak separation due to the highly asymmetrical structure of the filter. In all, the CP-DSC method is currently more preferable for a reliable determination of the pore-size mode in the filter paper. Developments in automated commercial LLP instruments will likely improve the quality of obtained data with this method.

**Figure 6:**
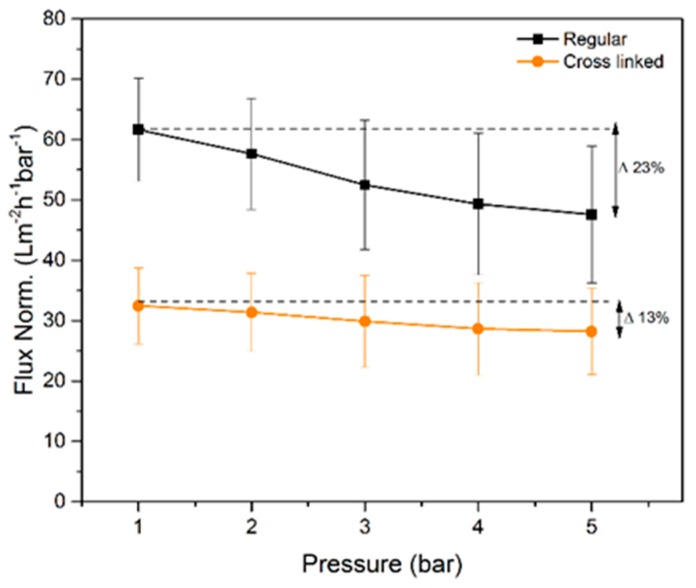
Normalized flow rate values measured for regular and cross-linked samples (*n* = 3). The delta values indicate the reduced flow rates per bar between 1 and 5 bar.

The measured normalized flux rates of water for the regular and cross-linked filter paper are presented in Figure 6. The regular filter paper exhibits higher normalized flux rates at all tested pressures compared to the cross-linked sample. The latter was expected due to the larger pore volume measured with NGSP and CP-DCS for the regular filter, i.e. smaller total pore volume results in lower flux values. The normalized flux decreased progressively with increased transmembrane pressure. The later indicates compression of the filter as the overhead pressure is increased as discussed previously [16]. Although both filters showed decrease in normalized flux values with increased pressure, the cross-linked filter paper was more robust and less prone to compression since only 13% reduction of normalized flux rate was observed between 1 and 5 bar. For the regular filter paper, the normalized flux rate reduction over the same pressure range was 22%. These results are well in line with the SEM cross-section images in Figure 3, which demonstrate a more densely packed structure for the as-prepared cross-linked filter paper.

**Figure 7:**
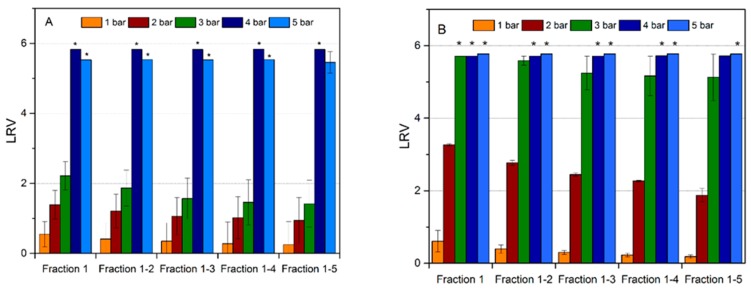
(**A**) MVM removal capability by mille-feuille filter at varying TM pressure, (**B**) MVM removal capability by cross-linked mille-feuille filter at varying TM pressure, (*) no infective viral particles detected in large-volume plating. TM: transmembrane.

To further characterize the performance of the filter, removal of worst-case model parvovirus, i.e. MVM (20 nm; nonenveloped) suspended in a 1 wt% bovine serum albumin (BSA) solution, was investigated using the regular and cross-linked samples. Figure 7 demonstrates the influence of the TM pressure on viral retention of the regular and cross-linked filter. No clogging was observed in any of the tested cases at any of the applied TM pressures. As it is seen for the regular filter in Figure 7A, virus retention was predominantly insignificant at TM pressures of 1–2 bar, while it was indicative, i.e. LRV < 2, at a filtration pressure of 3 bar. LRV increased successively with each pressure. At TM pressures of 4–5 bar, the virus removal capability was high, i.e. LRV ≥ 5 was achieved probably due to filter compaction at higher pressures. It should also be noted that for pressures ≤ 3 bar, the virus retention capability of the filter progressively decreased for each new fraction, yet this was not the case for tests performed at 4–5 bar. Figure 7B shows the virus removal results from the cross-linked filters. The cross-linked mille-feuille filters showed higher MVM removal capability compared to the regular samples. In particular, the virus removal capability was enhanced for all applied pressures. Furthermore, absence of any residual infective MVM particles (LRV ≥ 5) in LVP was recorded at 4–5 bar for all fractions. The most significant enhancement of virus removal capability was observed at 3 bar, wherein LRV ≥ 5 was observed for the cross-linked sample as compared to LRV < 2 for the regular filter.

It should be noted that the different methods did not show large differences in the pore size between filters but virus removal capability was greatly affected by the cross-linking and pressure gradient. The enhanced wet strength and compactness of the cross-linked filter in the wet state accounts for the observed increase in virus removal capability as discussed above in Figure 6. This is the first time the effect of varying TM pressure and cross-linking on MVM removal in the mille-feuille filter was shown. It should, however, be noted that varying LRVs at different TM pressures were also recorded in the past for other filters, e.g., for Planova BioEx [30]. It can be speculated that the cross-linking of the filter paper reduces the total pore volume of the filter and thereby restricts the free diffusive movement of virus particles in the three-dimensional pore space. The latter results in more robust virus removal properties. Clearly, further studies are necessary to understand the underlying mechanisms of virus capture during size-exclusion filtration in nanocellulose-based filter paper along the following lines: (i) understanding the effect of filter cross-linking on virus removal and throughput, and (ii) impact of cumulative virus load on observed LRVs over long-time bioprocessing.

## 4. Conclusions

In this work, comparative analysis of dry- and wet-porometry methods was performed to characterize regular and cross-linked virus removal filter paper using 3 independent methods. The results of the study suggest that wet-porometry methods yield a slightly larger pore size than that detected by NGSP. CP-DSC method was found particularly useful to characterize the PSD in the wet state. NGSP, CP-DSC, and LLP did not detect substantial pore-mode shift between regular and cross-linked filters. The effect of TM pressure on the efficiency of MVM removal was also investigated for the first time for regular and cross-linked filters. TM pressure of >3 bar was needed to achieve ≥4 LRV removal for noncross-linked samples. Cross-linking the cellulose nanofibers substantially improved the MVM removal efficiency at lower pressures enabling ≥4 LRV removal at 3–4 bar. This is particularly interesting since neither of the characterization methods could detect any change in the pore size for the cross-linked sample. In all, the results of this study highlight the complex nature of virus capture in size-exclusion filters, involving not only steric hindrance but also other parameters, such as hydrodynamic constraint at high pressures and also possibly weak-type surface interactions.

## Figures and Tables

**Table 1:** Summary of measured pore mode for Barrett-Jonyer-Halenda (BJH), LLP, and differential scanning calorimetry (DSC), the results are the average with ±SD.

Method	Pore Mode Regular (nm)	Pore Mode Cross-Linked (nm)
BJH	18.9 ± 1.4 ^a^	20.0 ± 2.3 ^a^
LLP	24.3 ± 2.5 ^b^	22.0 ± 1.4 ^b^
DSC	24.0 ± 0.8 ^c^	24.1 ± 0.6 ^c^

^a^*n* = 4, ^b^ *n* = 3, ^c^ *n* = 3.
